# Local Mucosal CO_2_ but Not O_2_ Insufflation Improves Gastric and Oral Microcirculatory Oxygenation in a Canine Model of Mild Hemorrhagic Shock

**DOI:** 10.3389/fmed.2022.867298

**Published:** 2022-04-28

**Authors:** Stefan Hof, Richard Truse, Lea Weber, Anna Herminghaus, Jan Schulz, Andreas P. M. Weber, Eva Maleckova, Inge Bauer, Olaf Picker, Christian Vollmer

**Affiliations:** ^1^Department of Anesthesiology, Duesseldorf University Hospital, Duesseldorf, Germany; ^2^Institute of Plant Biochemistry, Cluster of Excellence on Plant Sciences (CEPLAS), Heinrich-Heine-University Duesseldorf, Duesseldorf, Germany

**Keywords:** gastric microcirculation, μHbO_2_, hypercapnia, hyperoxia, hemorrhagic shock, mucosal barrier integrity

## Abstract

**Introduction:**

Acute hemorrhage results in perfusion deficit and regional hypoxia. Since failure of intestinal integrity seem to be the linking element between hemorrhage, delayed multi organ failure, and mortality, it is crucial to maintain intestinal microcirculation in acute hemorrhage. During critical bleeding physicians increase FiO_2_ to raise total blood oxygen content. Likewise, a systemic hypercapnia was reported to maintain microvascular oxygenation (μHbO_2_). Both, O_2_ and CO_2_, may have adverse effects when applied systemically that might be prevented by local application. Therefore, we investigated the effects of local hyperoxia and hypercapnia on the gastric and oral microcirculation.

**Methods:**

Six female foxhounds were anaesthetized, randomized into eight groups and tested in a cross-over design. The dogs received a local CO_2_-, O_2_-, or N_2_-administration to their oral and gastric mucosa. Hemorrhagic shock was induced through a withdrawal of 20% of estimated blood volume followed by retransfusion 60 min later. In control groups no shock was induced. Reflectance spectrophotometry and laser Doppler were performed at the gastric and oral surface. Oral microcirculation was visualized by incident dark field imaging. Systemic hemodynamic parameters were recorded continuously. Statistics were performed using a two-way-ANOVA for repeated measurements and *post hoc* analysis was conducted by Bonferroni testing (*p* < 0.05).

**Results:**

The gastric μHbO_2_ decreased from 76 ± 3% to 38 ± 4% during hemorrhage in normocapnic animals. Local hypercapnia ameliorated the decrease of μHbO_2_ from 78 ± 4% to 51 ± 8%. Similarly, the oral μHbO_2_ decreased from 81 ± 1% to 36 ± 4% under hemorrhagic conditions and was diminished by local hypercapnia (54 ± 4%). The oral microvascular flow quality but not the total microvascular blood flow was significantly improved by local hypercapnia. Local O_2_-application failed to change microvascular oxygenation, perfusion or flow quality. Neither CO_2_ nor O_2_ changed microcirculatory parameters and macrocirculatory hemodynamics under physiological conditions.

**Discussion:**

Local hypercapnia improved microvascular oxygenation and was associated with a continuous blood flow in hypercapnic individuals undergoing hemorrhagic shock. Local O_2_ application did not change microvascular oxygenation, perfusion and blood flow profiles in hemorrhage. Local gas application and change of microcirculation has no side effects on macrocirculatory parameters.

## Introduction

The potential for hemorrhage in trauma and surgical patients represents an ongoing concern for management (1) with an estimated 1.9 million deaths per year worldwide (2). With the exception of the traumatic event itself, exsanguination is the most frequent cause of immediate death (3) later on followed by multi organ dysfunction syndrome (MODS) as the leading cause of death among patients who die in intensive care units (4). In hemorrhagic shock severe blood loss causes an inadequate oxygen delivery to meet oxygen demand on a cellular level. Early recognition of hemorrhagic shock and immediate action to stop further bleeding are crucial, since the median time from onset of shock to death is 2 h (5). However, ischemia-reperfusion injury by restoration of blood flow may further aggravate tissue damage (6).

Therefore, as adjuncts to volume resuscitation, tissue hypoxia can be attenuated amongst others by increasing arterial oxygen content, restoration of organ perfusion, and reduction in local oxygen demand. Hyperoxia by increasing the fraction of inspired oxygen is effective to increase arterial oxygen content. However, excessive normobaric oxygen supplementation may have detrimental systemic effects because of enhanced oxidative stress and inflammation and hyperoxia-derived vasoconstriction resulting in reduced microvascular perfusion (7, 8). Permissive hypercapnia is common in critically ill patients and has been associated with augmented cardiac output and tissue oxygenation in experimental models of sepsis (9, 10) and hemorrhagic shock (11). On the other hand, hypercapnic acidosis exerts potent anti-inflammatory effects (12) and may even promote microbial growth (13), which could be detrimental in the context of sepsis. To limit systemic side effects local therapy regimes have been established in various organs and diseases, like inhaled nitric oxide in pulmonary hypertension (14).

The ischemic gut seems to be an important factor in the pathogenesis of MODS after traumatic hemorrhagic shock. In pathological conditions with reduced splanchnic perfusion, such as hemorrhagic shock, bacteria and gut ischemia provoke an intestinal inflammatory response, which leads to dysfunction of additional organs (15). Therefore, maintaining adequate gastrointestinal microcirculation is of utmost importance. Current strategies to prevent or reverse gut dysfunction focus on early enteral nutrition, which has been shown to improve intestinal barrier function, gastrointestinal immunity and resorptive function and to preserve gastrointestinal mucosal architecture (16). Moreover, because of its non-invasive accessibility, particularly the gastrointestinal tract is suitable for topical therapeutic interventions. For example, topical applied nitroglycerin and iloprost improved gastric oxygenation in a hemorrhagic shock model. In the same trial nitroglycerin improved intestinal barrier function (17). Likewise, local application of melatonin (18) and losartan (19) were able to ameliorate the decrease of gastric mucosal oxygenation in a state of acute hemorrhage. Systemic hypercapnia and hyperoxia proved to exert favorable effects in the context of hemorrhagic shock (11, 20). However, to date no data are available on the effects of local mucosal hyperoxia and hypercapnia on gastrointestinal microcirculatory oxygenation and perfusion in the presence of hemorrhage.

We hypothesized that local oxygen and carbon dioxide application modulates oral and gastric microcirculation and oxygenation in a mild model of hemorrhagic shock without adverse side effects on systemic variables.

Taken together, this study was designed to address the following questions:

(i) Does local CO_2_ or O_2_ increase gastric and oral oxygenation in a model of mild hemorrhagic shock?
(ii) Which impact has a local CO_2_- or O_2_-application on oral microcirculatory variables?
(iii) Are there any systemic side effects of local gas application expected, which could limit the use of local CO_2_ or O_2_ in critically ill patients?

## Materials and Methods

### Animals

The data were derived in a crossover design from repetitive experiments on six dogs (female foxhounds, weighing 28–36 kg) treated in accordance with the NIH guidelines for animal care. Experiments were performed with approval of the Local Animal Care and Use Committee (North Rhine-Westphalia State Agency for Nature, Environment, and Consumer Protection, Recklinghausen, Germany; ref. 84-02.04.2012.A152).

A slightly modified, well-established canine model of hemorrhagic shock was used as published previously (11). All animals were bred for experimental purposes and obtained from the animal research facility (ZETT, Zentrale Einrichtung für Tierforschung und wissenschaftliche Tierschutzaufgaben) of the Heinrich-Heine-University Duesseldorf. The animal husbandry took place in accordance with the European Directive 2010/63/EU and the National Animal Welfare Act. All animals were kept in kennel maintenance under the care of a keeper and with access to an outdoor area. The dogs were fed daily with dry food (Deukadog nature food lamb and rice, Deutsche Tiernahrung Cremer, Duesseldorf, Germany) and wet food (Rinti Gourmet Beef, Finnern, Verden, Germany). Prior to the experiments, access to food was withheld for 12 h with water *ad libitum* to ensure complete gastric depletion and to avoid changes in mucosal perfusion and oxygenation due to digestive activity. Each dog underwent every experimental protocol in a randomized order and served as its own control. The experiments were performed at least 3 weeks apart to prevent carryover effects. The experiments were performed under general anesthesia [induction of anesthesia with 4 mg⋅kg-1 propofol, maintenance with sevoflurane, end-tidal concentration of 3.0% (1.5 minimum alveolar concentration (MAC) for dogs)]. Following endotracheal intubation the dogs were mechanically ventilated [FiO_2_ = 0.3, VT = 12.5 ml⋅kg^–1^, a physiological tidal volume for dogs (21)], and the respiratory frequency adjusted to achieve normocapnia [end-expiratory carbon dioxide (etCO_2_) = 35 mmHg], verified by continuous capnography (Capnomac Ultima, Datex Instrumentarium, Helsinki, Finland). After taking each blood sample for blood gas analysis and determination of sucrose plasma levels, normal saline was infused three times the sampling volume to maintain blood volume. Throughout the experiments, the animals received less than 250 ml additional fluid replacement to avoid volume effects that could influence tissue perfusion and oxygenation. Following the intervention, the dogs were extubated as soon as they showed sufficient spontaneous breathing and protective reflexes. The animals remained under direct supervision of the laboratory personnel until complete recovery from anesthesia and first intake of wet food and water. After examination of all puncture sites and a detailed handover, the dogs were given back to their keepers in the animal research facility. No animal was sacrificed during or after the experiments.

### Measurements

#### Systemic Hemodynamic and Oxygenation Variables

The aorta was catheterized via the left carotid artery for continuous measurement of mean arterial pressure (MAP, Gould-Statham pressure transducers P23ID, Elk Grove, IL) and intermittent arterial blood gas analysis (Rapidlab 860, Bayer AG, Germany). Before participation in the study, both carotid arteries were externalized in all animals (22) to enable landmark guided arterial catheterization with low complication rates. Cardiac output (CO) was determined via transpulmonary thermodilution (PiCCO 4.2 non US, PULSION Medical Systems, Munich, Germany) at the end of each intervention. Systemic oxygen delivery (DO_2_) was calculated as the total oxygen content multiplied by cardiac output. Cardiac output and systemic oxygen delivery are related to the total body weight of each dog to ensure comparability between individuals. Heart rate (HR) was continuously measured by electrocardiography (Powerlab, ADInstruments, Castle Hill, Australia).

#### Mucosal Oxygenation and Perfusion

Microvascular oxygenation (μHbO_2_) and microvascular blood flow (μflow) of the gastric and oral mucosa were continuously assessed by tissue reflectance spectrophotometry and laser Doppler flowmetry (O_2_C, LEA Medizintechnik, Gieβen, Germany), respectively, as detailed previously (23). White light (450–1,000 nm) and laser light (820 nm, 30 mW) were transmitted to the tissue of interest via a microlightguide and the reflected light was analyzed. The wavelength-dependent absorption and overall absorption of the applied white light can be used to calculate the percentage of oxygenated hemoglobin (μHbO_2_) and the relative amount of hemoglobin (rHb). Due to the Doppler effect, magnitude and frequency distribution of changes in wavelength are proportional to the number of blood cells multiplied by the measured mean velocity (μvelo) of these cells. This product is proportional to flow and expressed in arbitrary perfusion units (aU). Hence, this method allows assessment and comparison of oxygenation and perfusion of the same region at the same time. Since light is fully absorbed in vessels with a diameter > 100 μm (24) only the microvascular oxygenation of nutritive vessels of the mucosa is measured. The biggest fraction of total blood volume is stored in venous vessels; therefore, mainly postcapillary oxygenation is measured which represents the critical partial pressure of oxygen (pO_2_) for ischemia (23). Two probes were used to meet the individual needs of the oral and gastric mucosal surface. According to the manufacturer’s recommendation reading were obtained by placing one flat lightguide probe in the mouth facing the buccal side of the oral mucosa (LF-2, 12 × 5.5 × 44.5 mm, LEA Medizintechnik GmbH, Gieβen) and a second flexible lightguide probe into the stomach (LM-10, outer diameter 2.6 mm, LEA Medizintechnik GmbH, Gieβen) via an orogastric silicone tube facing the gastric mucosa. Continuous evaluation of the signal quality throughout the experiments and the immediate comparison between oral and gastric spectroscopy allows verification of the correct probe position during the experiments. The μHbO_2_ and μflow values reported are 5 min means (150 spectra, 2 s each) of the respective intervention under steady state conditions. The non-traumatic access to the gastric mucosa allows the determination of mucosal microcirculation without the need of surgical stress. This is particularly desirable regarding the marked alterations that surgical stress exerts on splanchnic circulation. Under these circumstances reflectance spectrophotometry reliably detects even clinically asymptomatic reductions in μHbO_2_ and highly correlates with the morphologic severity and extent of gastric mucosal tissue injury (25).

#### Mucosal Microcirculation—Videomicroscopy

Microcirculatory perfusion of the oral mucosa was assessed intermittently by incident dark field (IDF) -imaging (CytoCam, Braedius Medical, Huizen, Netherlands) as described elsewhere (26). Illumination was provided by light emitting diodes (LED) at a wavelength of 530 nm, the isosbestic point for deoxy- and oxyhemoglobin, and directed toward the oral mucosa. The reflected and scattered light is filtered and visualizes red blood cells in capillaries. All videos were obtained by the same investigator. Videos were saved anonymized for blinded analysis. To assess the predominant blood flow characteristics, a well-established semiquantitative scoring method, the microcirculatory flow index (MFI), was used to characterize microcirculatory flow as “no flow,” “intermittent flow,” “sluggish flow,” and “continuous flow” (27). Many clinical trials reported critical illness to be associated with heterogeneous blood flow patterns, which may assume microvascular shunting to be responsible for an impaired oxygen delivery in this collective (28, 29). Therefore, we calculated the heterogeneity index (HGI) as the difference between the highest and the lowest value achieved in the semiquantitative scoring system and divided it through the averaged microvascular flow index to determine spatial flow heterogeneity. To assess vessel density, the total vessel density (TVD), including perfused and non-perfused microvessels, and perfused vessel density (PVD), including perfused microvessels only, were analyzed using the appropriate software (MicroCirculation Analysis software, Braedius Medical, Huizen Netherlands) (30). The PVD/TVD ratio is used to express the proportion of perfused vessels (PPV). Only vessels with a diameter smaller than 20 μm are included in the analysis. Thus, the PVD represents the functional capillary density (FCD), which is considered to be the main determinant of microcirculatory blood supply and oxygen diffusion distance (31).

#### Intestinal Barrier Function

The disaccharide sucrose (D-Sucrose, Carl Roth, Karlsruhe, Germany) was infused into the stomach (1.66 g⋅kg^–1^ body weight) via an orogastric tube prior to the induction of hemorrhagic shock. Under physiological conditions, sucrose does not pass intact gastric mucosa. After being transported from the gastric region into the small intestine, ingested sucrose is rapidly degraded by sucrose-isomaltase into monosaccharides. Therefore, intact sucrose cannot be found in blood plasma under physiological conditions. However, under compromising conditions such as shock, barrier function is impaired, and sucrose can pass the gastric mucosal barrier into the plasma, where it does not undergo any enzymatic reaction. Sucrose plasma levels can therefore be used to assess gastric mucosal barrier function (32). Blood samples were collected under baseline conditions and during hemorrhagic shock. The collected samples were prepared as previously described (17). Briefly, blood plasma was separated via centrifugation (Rotina 420R, Hettich Zentrifugen, Mülheim a. d. R., Germany). Cold extraction mixture containing 10 μM ribitol, aceton and isopropanol (all from Carl Roth) was freshly prepared at a ratio of 2:1 and added to each probe. The supernatant was taken after the total probe was mixed and centrifugated again, denitrogenized and stored at −80^°^C. After completion of the experiments, extracts were dried using a speed vacuum concentrator (RVC 2-25 CDplus, Christ) for 3.5 h and obtained pellets were resuspended in 50 μl water (HPLC grade, Fisher Chemical). Subsequently, samples were purified using activated charcoal followed by filtration. After addition of a pinch of activated charcoal (Carl Roth; Karlsruhe, Germany), samples were vortexed briefly and incubated for a few minutes before centrifugation at 16,000 g and 8^°^C for 7 min. Collected supernatants were transferred to nylon-membrane centrifugal filters with pore size of 0.2 μm (VWR, 516-0233). Follow-throughs recovered after the centrifugation at room temperature at maximal speed for 2 min were stored at −80^°^C until analysis. Concentration of glucose-6-phosphate, glucose, fructose and saccharose was determined enzymatically using a modification of protocol by Stitt (33). Aliquots of 15 μl sample or 10 μl standard (ranging from 0 to 25 nM) were added to 100 mM HEPES-NaOH (pH 7.5) with 10 mM MgCl2, 3 mM NADP + (disodium salt, Carl Roth, Karlsruhe, Germany) and 6 mM ATP (disodium salt hydrate, Sigma-Aldrich, St. Louis, MO, United States). After reading absorbance at 340 nm for 15 min, 2.1 U glucose-phosphate dehydrogenase (10127671001, Roche, Basel, Switzerland), 13.5 U hexokinase (11426362001, Roche, Basel, Switzerland), 1.5 U glucose-6-phosphate isomerase (10127396001) and approximately 7 U invertase (I4504-1G, Sigma-Aldrich. St. Louis, MO, United States) were added subsequently, with absorbance of individual reactions being read until stability using microplate reader (Synergy H1, BioTek, Winooski, VT, United States).

### Experimental Protocol

**FIGURE 1:**
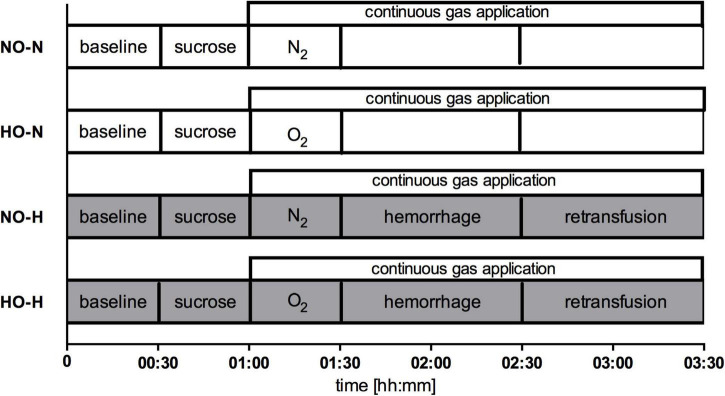
Experimental protocol set 1: Continuous application of N_2_ under normoxia (NO) or hyperoxia with application of O_2_ (HO) before and during physiological (-N) or hemorrhagic (-H) condition. In shock groups 1 h of hemorrhage was followed by retransfusion, while gas application was conducted until the termination of the experimental protocol in all groups.

**FIGURE 2:**
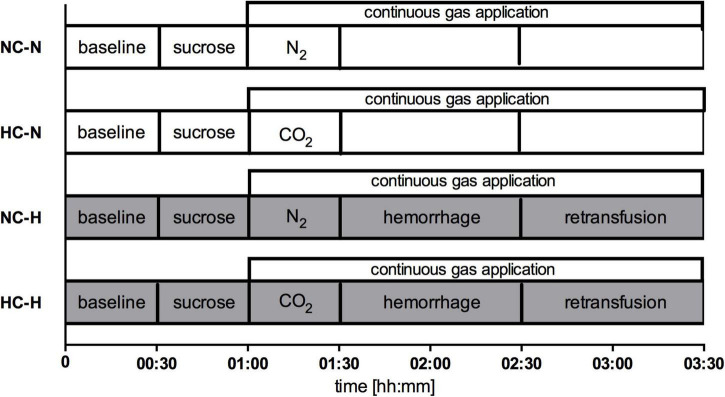
Experimental protocol set 2: Continuous application of N_2_ under normocapnia (NC) or hypercapnia with application of CO_2_ (HC) before and during physiological (-N) or hemorrhagic (-H) condition. In shock groups 1 h of hemorrhage was followed by retransfusion, while gas application was conducted until the termination of the experimental protocol in all groups.

The six dogs passed through two sets of experiments, each consisting of a normovolemic and a hypovolemic interventional group with their respective normoxic or normocapnic control groups. In the first set, effects of local oxygen insufflation were analyzed with and without hemorrhagic shock (Figure 1). In the second set, carbon dioxide was insufflated during both conditions (Figure 2). In both experimental setup’s nitrogen (N_2_) was insufflated during physiologic and hemorrhagic conditions as control. After instrumentation, 30 min were allowed to establish steady state conditions and baseline values were recorded before the animals were randomized to the respective protocol. Steady state conditions were defined as stability of hemodynamic variables (heart rate, mean arterial pressure) as well as ventilation parameters (end-tidal CO_2_, end-tidal sevoflurane concentration, inspiratory oxygen fraction).

First set of experiments:

- NO-N: Normoxia during normovolemia
- HO-N: Local gastric and oral hyperoxia during normovolemia
- NO-H: Normoxia during mild hemorrhagic shock
- HO-H: Local gastric and oral hyperoxia during mild hemorrhagic shock

Second set of experiments:

- NC-N: Normocapnia during normovolemia
- HC-N: Local gastric and oral hypercapnia during normovolemia
- NC-H: Normocapnia during mild hemorrhagic shock
- HO-H: Local gastric and oral hypercapnia during mild hemorrhagic shock

#### Local Oxygen and Carbon Dioxide Application

To study the effects of local oxygen and carbon dioxide insufflation, 30 min after application of the sugar solution, 15 ml/min of 100% oxygen in hyperoxic groups or 100% carbon dioxide in hypercapnic groups was directed to the gastric mucosal via a multi-lumen orogastric tube and to the oral mucosa via a custom-made retainer. Both devices were manufactured in our institute. The multi-lumen orogastric tube, with its three distal orifices on the same level (consisting of three suction catheters, 80 cm, CH18, Dahlhausen, Cologne, Germany) as well as the custom-made retainer were placed without further trauma on the mucosal surfaces. The distal orifice of the tube as well as the gas-leading catheters of the retainer were in close proximity to the O_2_C measuring probes (Supplementary Figure 1). The abdominal circumference was measured intermittently to exclude overinflation. All variables were recorded for the next 2.5 h. As time control experiments, 15 ml/min of pure nitrogen (groups NO-N and NO-H/groups NC-N and NC-H) was directed to the gastric and oral mucosa for 2.5 h.

#### Induction of Hemorrhagic Shock

Hemorrhagic shock was induced by removing 20% of the estimated total blood volume via a large bore intravenous cannula in a peripheral vein and the arterial catheter (i.e., 16 ml⋅kg^–1^ of whole blood) over 5 min. According to Advanced Trauma Life Support this model represents a class II shock (blood loss 15–30%) (34). The extracted blood was heparinized, stored and 60 min later retransfused using an infusion set with a 200 μm filter. Protamine was injected i.v. in a ratio of protamine to heparin as 1:2.

### Statistical Analysis

Data for analysis were obtained during the last 5 min of baseline and intervention periods under steady-state conditions. All data are presented as absolute values of mean ± standard error (mean ± SEM) for six dogs. Normal data distribution was assessed in Q-Q-plots (IBM SPSS Statistics, International Business Machine Corp., United States). Differences within the groups and between the groups were tested using a two-way analysis of variance for repeated measurements (ANOVA) and a Bonferroni test as *post-hoc* test (GraphPad Prism version 6.05 for Windows, GraphPad Software, La Jolla California United States). Microvascular flow index and heterogeneity index as semiquantitative scores generally require non-parametric analysis. Multiple measurements with five videos à four squares per measurement approximate a normal data distribution and enable metric statistical testing. Therefore, we used Q-Q-plots, two-way ANOVA and Bonferroni *post-hoc* test for statistical analysis, too. An à priori power analysis (G*Power Version 3.1.9.2) (35) revealed a power of 0.85 for detection of differences between the different groups with *n* = 6 in 4 groups, repeated measurements, α < 0.05 and η^2^ of 0.5 (calculated from previous experiments).

## Results

### Effect of Local Hypercapnia and Hyperoxia During Physiological Conditions

In anesthetized, ventilated dogs during otherwise physiological conditions, neither local O_2_- nor CO_2_-insufflation modified gastric or oral microcirculatory oxygenation and perfusion compared to the control groups (Table 1 and Supplementary Table 1). Further, local CO_2_-insufflation did not modify p_*a*_CO_2_ and systemic pH-values. In detail, p_*a*_CO_2_ increased from 35.5 ± 0.6 mmHg to a maximum of 37.1 ± 0.8 mmHg in the CO_2_ treated group, which was not different from the normocapnic control group, where p_*a*_CO_2_ increased during the experiment from 34.4 ± 0.3 mmHg to a maximum of 36.6 ± 0.9 mmHg. There was no difference between the respiratory rate in hypercapnic and normocapnic animals. Cardiac output and DO_2_ were significantly lower in the hypercapnic group compared to the control group. However, this effect already exists during baseline conditions, remained throughout the experiment independent of the intervention and could be followed back to one individual (Table 2). The presentation of CO and DO_2_ as the difference of baseline values demonstrates no effect in both parameters after CO_2_ -insufflation. No differences between the CO_2_ treated group and the control group were detected in MAP, HR and SVR.

**TABLE 1:** Microcirculatory variables in normo- (NC) and hypercapnic (HC) animals during physiological (-N) or hemorrhagic (-H) conditions.

Variables	Group	00:30 h			01:00 h				01.30 h				02:00 h				02:30 h				03:00 h				03:30 h			
Gastric μHbO_2_ (%)	NC-N	83	±	4	84	±	3		79	±	3		76	±	3		73	±	3		79	±	5		78	±	6	
	HC-N	77	±	2	77	±	1		80	±	4		78	±	4		76	±	3		77	±	3		79	±	2	
	NC-H	77	±	2	79	±	2		79	±	3		38	±	4	**#**	52	±	6	**#**	75	±	6		80	±	3	
	HC-H	83	±	3	83	±	2		85	±	2		52	±	8	#,*	57	±	8	**#**	81	±	4		82	±	3	
Gastric μflow (aU)	NC-N	153	±	10	268	±	38	**#**	260	±	26	**#**	245	±	31	**#**	266	±	42	**#**	264	±	22	**#**	237	±	35	
	HC-N	198	±	31	176	±	29	*	223	±	32		233	±	33		204	±	25		239	±	18		250	±	38	
	NC-H	145	±	17	172	±	19		177	±	24		171	±	19		160	±	22		185	±	30		212	±	31	
	HC-H	177	±	22	180	±	27		252	±	38		229	±	40		262	±	74	*	239	±	46		216	±	35	
Oral μHbO_2_ (%)	NC-N	85	±	3	83	±	3		84	±	2		81	±	1		83	±	2		83	±	2		83	±	2	
	HC-N	82	±	2	82	±	2		83	±	2		84	±	2		81	±	1		79	±	2		86	±	2	
	NC-H	82	±	2	79	±	2		82	±	1		36	±	4	**#**	45	±	5	**#**	78	±	6		93	±	2	**#**
	HC-H	83	±	2	81	±	3		86	±	3		54	±	4	#,*	63	±	5	#,*	79	±	4		90	±	2	
Oral μflow (aU)	NC-N	143	±	24	163	±	35		117	±	9		144	±	34		131	±	15		137	±	20		129	±	27	
	HC-N	128	±	14	121	±	13		168	±	31		185	±	27		146	±	35		162	±	31		143	±	21	**#**
	NC-H	94	±	18	121	±	36		110	±	26	*	50	±	17		38	±	9		109	±	18		191	±	29	
	HC-H	126	±	21	132	±	31		195	±	38		58	±	16		80	±	18		163	±	41		179	±	28	
MFI	NC-N	3	±	0	2.8	±	0.1		2.9	±	0.1		2.9	±	0		2.9	±	0.1		2.9	±	0.1		2.9	±	0.1	
	HC-N	3	±	0.7	2.9	±	1.1		2.9	±	1.4		2.9	±	1.4		2.9	±	1.5		2.7	±	2		2.8	±	1.9	
	NC-H	2.8	±	0.1	2.9	±	0		2.9	±	0.1		0.8	±	0.2	#,*	1.4	±	0.2	#,*	2.8	±	0.2		3	±	0	
	HC-H	2.9	±	0.1	2.9	±	0.1		2.9	±	0.1		1.3	±	0	**#**	2	±	0.3	**#**	2.7	±	0.1		2.9	±	0.1	
HGI	NC-N	0.7	±	0.1	1.6	±	0.1		1.1	±	0.1		1.4	±	0.1	**#**	1.1	±	0.1		1.1	±	0.1		0.4	±	0.1	
	HC-N	0.7	±	0.1	1.1	±	0.1		1.4	±	0.1		1.4	±	0.1		1.5	±	0.2		2	±	0.2		1.9	±	0.2	
	NC-H	1.5	±	0.1	1.4	±	0.1		1.4	±	0.1		16.7	±	0.5	**#**	11.6	±	0.3	**#**	3.1	±	0.3		0.4	±	0.1	
	HC-H	1.1	±	0.1	1.4	±	0.1		1.4	±	0.1		14.1	±	0.3	**#**	6.7	±	0.2	#,*	2.3	±	0.1		1.1	±	0.1	
TVD (mm/mm^2^)	NC-N	19	±	0	19	±	1		19	±	1		19	±	1		20	±	1		19	±	0		19	±	1	
	HC-N	19	±	1	19	±	0.5		19	±	1		19	±	1		18	±	1		18	±	0		18	±	0	
	NC-H	18	±	0	19	±	0.9		19	±	1		14	±	2	**#**	15	±	1	**#**	19	±	1		21	±	1	**#**
	HC-H	19	±	0	18	±	0.7		19	±	1		14	±	1	**#**	16	±	1	**#**	18	±	1		19	±	1	
PVD (mm/mm^2^)	NC-N	11	±	1	10	±	1.8		11	±	1		11	±	1		11	±	1		10	±	2		9	±	2	
	HC-N	13	±	1	12	±	1.1		12	±	1		12	±	1		11	±	1		9	±	1	**#**	10	±	0	
	NC-H	10	±	1	11	±	1.4		11	±	1		3	±	1	**#**	4	±	1	**#**	11	±	1		13	±	1	**#**
	HC-H	12	±	1	9	±	0.9		12	±	1		5	±	1	**#**	6	±	1	**#**	9	±	1		11	±	1	

Microvascular oxygenation (μHbO_2_) in percentage (%) and microvascular flow (μflow) in arbitrary units (aU) were measured at the gastric and oral surface using reflectance spectrophotometry and laser Doppler flowmetry. Microvascular flow index (MFI), heterogeneity index (HGI), total vessel density (TVD) in (mm/mm^2^) and PVD in (mm/mm^2^) were evaluated by incident dark field imagine. One hour of acute hemorrhage is marked gray. Data are presented as mean ± SEM for n = 6 dogs (MFI and HGI n = 5). ^#^p < 0.05 vs. baseline, *p < 0.05 vs. normoxic control group. 2-way ANOVA for repeated measurements followed by Bonferroni post-hoc test.

**TABLE 2:** Macrocirculatory variables and blood gas analysis in normo- (NC) and hypercapnic (HC) animals during physiological (-N) or hemorrhagic (-H) conditions.

Variables	Group	00:30 h			01:00 h				01.30 h				02:00 h				02:30 h				03:00 h				03:30 h			
DO_2_ (ml/kg/min)	NC-N	16	±	1	16	±	1		16	±	1		16	±	1		16	±	1		16	±	1		16	±	1	
	HC-N	15	±	1	14	±	0	*	14	±	0	*	14	±	0	*	14	±	0	*	14	±	1	*	14	±	1	*
	NC-H	14	±	1	14	±	1		14	±	1		7	±	1	**#**	8	±	1	**#**	14	±	1		15	±	1	
	HC-H	15	±	0	14	±	0		14	±	1		7	±	1	**#**	8	±	1	**#**	14	±	1		15	±	1	
CO (ml/kg/min)	NC-N	99	±	7	95	±	8		94	±	7		95	±	6		95	±	7		96	±	5		94	±	6	
	HC-N	88	±	2	85	±	2	*	85	±	2	*	82	±	2	*	84	±	2	*	8	±	3	*	82	±	2	*
	NC-H	87	±	4	83	±	4		82	±	4		45	±	3	**#**	51	±	3	**#**	87	±	4		92	±	5	
	HC-H	87	±	1	85	±	2		81	±	2		43	±	4	**#**	51	±	5	**#**	86	±	3		89	±	5	
MAP (mmHg)	NC-N	64	±	2	67	±	3		69	±	3		69	±	3		69	±	3		69	±	3		69	±	3	
	HC-N	61	±	2	66	±	3		68	±	3		67	±	3	**#**	70	±	4	**#**	70	±	4	**#**	70	±	3	**#**
	NC-H	65	±	2	69	±	4		68	±	3	**#**	50	±	3		56	±	2	**#**	87	±	3	**#**	76	±	5	**#**
	HC-H	65	±	1	66	±	2		69	±	2		50	±	2	**#**	57	±	1	**#**	85	±	4	**#**	72	±	6	**#**
pH	NC-N	7.39	±	0.01	7.38	±	0.01		7.37	±	0.01	**#**	7.36	±	0.01	**#**	7.36	±	0.01	**#**	7.36	±	0.01	**#**	7.35	±	0.0	**#**
	HC-N	7.39	±	0.01	7.38	±	0.01		7.38	±	0.01		7.38	±	0.01	*	7.37	±	0.01	**#**	7.37	±	0.01	**#**	7.38	±	0.0	*
	NC-H	7.4	±	0.01	7.39	±	0.01		7.39	±	0.01		7.32	±	0.01	**#**	7.33	±	0.01	**#**	7.37	±	0.01	**#**	7.37	±	0.0	**#**
	HC-H	7.4	±	0.01	7.39	±	0.01		7.39	±	0.01	**#**	7.33	±	0.01	**#**	7.32	±	0.01	**#**	7.37	±	0.01	**#**	7.37	±	0.0	**#**
p_*a*_CO_2_ (mmHg)	NC-N	34	±	0	35	±	0		35	±	1		36	±	0	**#**	36	±	1	**#**	37	±	1	**#**	36	±	1	**#**
	HC-N	36	±	1	36	±	0		36	±	0		37	±	0		37	±	1	**#**	37	±	1	**#**	36	±	1	
	NC-H	35	±	1	36	±	1		36	±	1		40	±	1	**#**	40	±	1	**#**	36	±	1		37	±	1	
	HC-H	35	±	1	36	±	1		36	±	1		40	±	1	**#**	41	±	1	**#**	37	±	1	**#**	36	±	1	**#**
BE (mmol/l)	NC-N	−3.7	±	0.34	−3.9	±	0.26		−4.1	±	0.3		-4.6	±	0.37	**#**	-4.8	±	0.51	**#**	−4.6	±	0.52	**#**	−4.8	±	0.45	**#**
	HC-N	−3.1	±	0.23	−3.2	±	0.17		−3.2	±	0.2	*	-3.2	±	0.32	*	-3.4	±	0.34	*	−3.5	±	0.39	*	−3.6	±	0.44	*
	NC-H	−2.8	±	0.14	−3	±	0.12		−3.2	±	0.12		-4.7	±	0.24	**#**	-4.6	±	0.25	**#**	−3.7	±	0.22	**#**	−3.6	±	0.25	**#**
	HC-H	−2.7	±	0.33	−2.9	±	0.22		−3.3	±	0.21		-4.9	±	0.19	**#**	-4.7	±	0.17	**#**	−3.9	±	0.21	**#**	−4	±	0.12	**#**
Lactate (mmol/l)	NC-N	1.1	±	0.2	1.3	±	0.2		1.6	±	0.2		2	±	0.3	**#**	2.3	±	0.4	**#**	2.2	±	0.4	**#**	2.3	±	0.4	**#**
	HC-N	1.1	±	0.1	1.2	±	0.2		1.2	±	0.1		1.2	±	0.1		1.3	±	0.1		1.3	±	0.1		1.4	±	0.1	
	NC-H	0.9	±	0.1	1.1	±	0.1		1.2	±	0.1		1.3	±	0.1		1.3	±	0.1		1.2	±	0.1		1.2	±	0.1	
	HC-H	0.9	±	0.2	1.1	±	0.2		1.4	±	0.2		1.6	±	0.2	**#**	1.5	±	0.2	**#**	1.5	±	0.3		1.6	±	0.3	**#**
Resp. rate (1/min)	NC-N	15	±	2	15	±	2		15	±	2		16	±	2		17	±	2		17	±	2	**#**	17	±	2	
	HC-N	14	±	1	14	±	1		15	±	1		15	±	1	*	16	±	1	*	16	±	2	#,*	16	±	1	#,*
	NC-H	14	±	1	14	±	1		15	±	1		13	±	1		15	±	1		17	±	1	**#**	18	±	2	**#**
	HC-H	15	±	1	14	±	1		16	±	1		14	±	1		15	±	1		18	±	2	**#**	18	±	1	**#**
p_*a*_O_2_ (mmHg)	NC-N	153	±	2	152	±	2		155	±	4		155	±	4		155	±	3		153	±	3		161	±	4	
	HC-N	151	±	5	152	±	2		153	±	1		156	±	2		156	±	2		154	±	3		160	±	2	**#**
	NC-H	149	±	3	156	±	5		162	±	3	**#**	142	±	2		150	±	4		164	±	2	**#**	156	±	44	*
	HC-H	153	±	5	154	±	4		159	±	3		147	±	3		149	±	2		167	±	3	**#**	166	±	2	**#**

Systemic oxygen delivery (DO_2_) in (ml/kg/min), cardiac output (CO) in (ml/kg/min), mean arterial pressure (MAP) in (mmHg), ph-values, arterial carbon dioxide pressure (p_a_CO_2_) in (mmHg), base excess (BE) in (mmol/l), lactate concentration in (mmol/l) and arterial oxygen pressure (p_a_O_2_) in (mmHg). One hour of acute hemorrhage is marked gray. Data are presented as mean ± SEM for n = 6 dogs. ^#^p < 0.05 vs. baseline, *p < 0.05 vs. normocapnic control group. 2-way ANOVA for repeated measurements followed by Bonferroni post-hoc test.

Under physiological conditions neither macrocirculatory parameters nor blood gases were affected by local oxygen application compared to their control group. Further, animals did not show any differences in hemodynamics and systemic oxygen delivery linked to local hyperoxia (Supplementary Table 2).

### Effect of Local Hypercapnia and Hyperoxia During Hemorrhagic Conditions

#### Gastric Microcirculation

**FIGURE 3:**
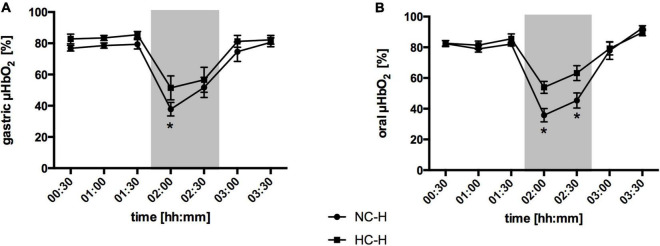
Effect of local carbon dioxide application on microvascular oxygenation: Microvascular oxygenation (μHbO_2_) in percentage (%) at the gastric **(A)** and oral **(B)** mucosa under hemorrhagic conditions. The animals received a continuous gas application with either N_2_ to maintain local normocapnia (NC-H) or CO_2_ for local hypercapnia (HC-H) followed by hemorrhage and retransfusion. One hour of acute hemorrhage is marked gray. Data are presented as mean ± SEM for *n* = 6 dogs; *p < 0.05 vs. control group (NC-H), 2-way ANOVA for repeated measurements followed by the Bonferroni *post-hoc* test.

**FIGURE 4:**
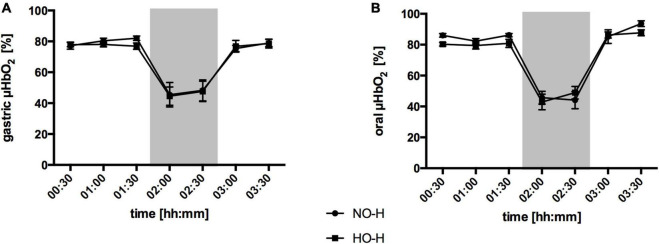
Effect of local oxygen application on microvascular oxygenation: Microvascular oxygenation (μHbO_2_) in percentage (%) at the gastric **(A)** and oral **(B)** mucosa. The animals received a continuous gas application with either N_2_ to maintain local normoxia (NO-H) or O_2_ for local hyperoxia (HO-H) followed by hemorrhage and retransfusion. One hour of acute hemorrhage is marked grey. Data are presented as mean SEM for *n* = 6 dogs.

Hemorrhagic shock led to a pronounced decrease in gastric μHbO_2_ in the normocapnic control group from 77 ± 2 to 38 ± 4% (Figure 3A) and in the normoxic control group from 77 ± 2 to 45 ± 7% (Figure 4A). Local hypercapnia, but not hyperoxia ameliorated the shock-induced decrease in gastric μHbO_2_ and led to a reduction from 83 ± 3 to 52 ± 8% in the early course of shock. Gastric insufflation of oxygen did not affect regional microcirculatory oxygenation under hypovolemic conditions. Microvascular perfusion was not altered in animals receiving hyperoxia or hypercapnia compared to their respective control group during hemorrhage (Table 1 and Supplementary Table 1). After resuscitation, μHbO_2_ baseline values were reached in all groups.

#### Oral Microcirculation

Similar to the gastric microcirculation, the induction of a hemorrhagic shock led to a significant reduction in oral μHbO_2_ in the normocapnic control group from 82 ± 2 to 36 ± 4% (Figure 3B) and in the normoxic control group from 86 ± 2 to 46 ± 6% (Figure 4B). The decrease in oral μHbO_2_ was diminished to 54 ± 4% in the hypercapnic group. This effect continued over the 60 min shock period. Mucosal hyperoxia had no effect on oral microcirculatory oxygenation.

**FIGURE 5:**
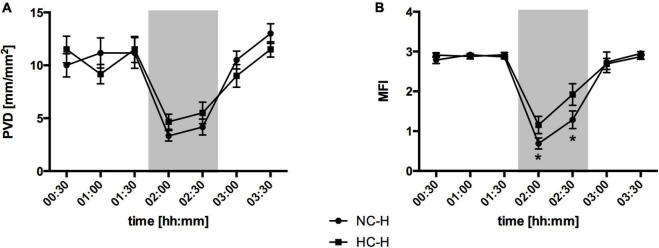
Effect of local carbon dioxide application on microvascular perfusion variables: Perfused vessel density (PVD) in (mm/mm^2^) **(A)** and Microvascular flow index (MFI) **(B)** at oral mucosa under hemorrhagic conditions. The animals received a continuous gas application with either N_2_ to maintain local normocapnia (NC-H) or CO_2_ for local hypercapnia (HC-H) followed by hemorrhage and retransfusion. One hour of acute hemorrhage is marked gray. Data are presented as mean SEM for *n* = 6 dogs; *p < 0.05 vs. control group (NC-H), 2-way ANOVA for repeated measurements followed by the Bonferroni *post-hoc* test.

Absolute μflow measured by laser Doppler and the perfused vessel density evaluated by videomicroscopy did not differ between the interventional and the control groups, there was a profound difference in the predominant microvascular flow profiles in hemorrhagic animals under hypercapnic conditions (Figure 5): The microvascular flow index decreased in the normocapnic control group from 2.8 ± 0.1 to 0.8 ± 0.2. Local hypercapnia ameliorated the decrease from 2.9 ± 0.1 to 1.3 ± 0. Due to reasons of imaging quality, IDF-recording of one dog receiving hypercapnia under otherwise physiological conditions had to be excluded from the analysis in accordance with common recommendations on the assessment of sublingual microcirculation (36). Therefore, microvascular flow index is reported for *n* = 5 in the experimental setup investigating effects of local CO_2_. Animals that received oxygen supply did not show comparable changes in a hemorrhagic state.

Heterogeneity index increased from 0.3 ± 0.1 to 2.8 ± 0.5 in normocapnic individuals and from 0.4 ± 0 to 1.6 ± 0.3 in normoxic animals when hemorrhage was induced. Neither CO_2_- (2.5 ± 0.7) nor O_2_-application (1.1 ± 0.3) could significantly diminish spatial flow heterogeneity during early hemorrhage. Despite, in the late course of shock heterogeneity index was higher in the normocapnic animals (1.9 ± 0.3) than in the hypercapnic animals (1.1 ± 0.2). In normocapnic animals total vessel density and perfused vessel density decreased when hemorrhage was induced (Table 1). Local hyperoxia had no effect on the changes of the structural microcirculation during hemorrhagic shock (Supplementary Table 1).

### Intestinal Barrier Function

Plasma levels of sucrose increased in normocapnic animals from 1 ± 0.4 pmol/μl to 1.8 ± 1.3 pmol/μl and in hypercapnic animals from 0.2 ± 0.1 pmol/μl to 1.8 ± 1.1 pmol/μl during hemorrhage without any statistically significant difference between the two interventional groups. Moreover, sucrose concentration increased in normoxic and hyperoxic experiments independent of hemodynamic conditions. All experimental groups showed a wide range of inter-individual variability (Supplementary Table 3).

### Global Hemodynamics and Ventilation

DO_2_ decreased during hemorrhage in all groups irrespective of the intervention (Table 2 and Supplementary Table 2). The decrease of DO_2_ was based on a similar decrease in cardiac output. After retransfusion of shed blood, DO_2_ was restored to baseline values in all experimental groups. Similar changes occurred in cardiac output and mean arterial blood pressure. Neither local CO_2_- nor O_2_-insufflation affected systemic hemodynamic parameters and did not modulate oxygen delivery or oxygen content of arterial blood (P_*a*_O_2_).

The arterial carbon dioxide concentration increased during shock paralleled by decreased pH-values without any effect of local gas application. The respiratory frequency was adjusted to maintain an endtidal carbon dioxide concentration of 35 mmHg. Local gas application had no effect on respiratory rate. Further, mucosal application of CO_2_ and O_2_ had no effect on systemic metabolic variables during hemorrhage. Base excess and pH were equally reduced and p_*a*_CO_2_ was increased in all groups. During hemorrhagic shock, no clinically relevant alterations in lactate plasma levels were observed (Table 1 and Supplementary Table 1).

## Discussion

The aim of our study was to analyze the effect of a local O_2_- and CO_2_-application on regional microcirculation and systemic hemodynamic variables during physiologic conditions and during a mild hemorrhagic shock. Whether our results can be transferred to more severe shock conditions remains unclear and has to be addressed in further studies due to the minimal invasive experimental setting. Likewise, additional invasive measurements were prohibited in this reversible shock model. However, the shock was able to induce micro- and macrocirculatory impairment and can be declared to be reversible since retransfusion of the shed blood was able to re-establish baseline values in the course of experiments. The main findings are:

1) Local CO_2_ but not O_2_ increases oral and gastric microvascular oxygenation during a mild hemorrhagic shock.
2) Increases of oral microvascular oxygenation in hypercapnic individuals are associated with changes in microcirculatory flow characteristics without changing total microvascular blood flow.
3) Local O_2_- and CO_2_-application do not exert effects on micro- and macrocirculatory parameters under physiological conditions.

Molecular oxygen and carbon dioxide dominate the two different sites of cellular respiration and energy metabolism. O_2_ is crucial to enable aerobic phosphorylation, whereas CO_2_ is one main product after energy generation in the mitochondria. CO_2_ needs an adequate tissue perfusion to be eliminated from the microcirculation, therefore local carbon dioxide pressure and pCO_2_-gap are able to detect states of severe tissue hypoperfusion. Since a sufficient tissue oxygenation depends on an adequate tissue perfusion, local carbon dioxide pressure and pCO_2_-gap may be indirect parameters of tissue oxygenation as well (37). Consequently, the ratio of veno-arterial pCO_2_-gradient to the difference in arterio-venous O_2_-content is reported to detect hypoxic states in critically ill patients (38, 39).

The intent to examine both gases as potential therapeutic options in experimental hemorrhagic shock is based on distinct observations. On the one hand, identifying severe hemorrhage as a state of compromised oxygen delivery leads physicians to perform a bundle of therapeutic interventions including blood and fluid replacement, vasopressor application, and an increase of inspiratory oxygen fraction to obtain total oxygen content. On the other hand, the generation of reactive oxygen species may further harm the injured tissue (40). In our experiments the additional application of local oxygen was not able to improve microvascular oxygenation. Since hyperoxia is reported to induce precapillary vasoconstriction (7), one may conclude that hyperoxic vasoconstriction might counteract the improvement of microvascular oxygenation through an increase of arterial oxygen concentration. Nevertheless, neither total microvascular flow assessed by laser flowmetry, nor microvascular flow characteristics were altered by local oxygen application. Further, structural microcirculation evaluated by IDF-imaging did not differ between hyperoxic and normoxic individuals. With no changes in total blood flow and structural microcirculation the question arises why hyperoxia does not lead to an increase of microcirculatory oxygenation. One aspect we cannot derive from microcirculatory measurements is the mitochondrial oxygen utilization. Oxygen utilization and mitochondrial efficiency are of large interest since oxidative respiration is acknowledged as the final goal of therapeutic interventions to optimize oxygen delivery in critical illness (41). In pulmonary endothelial cells hyperoxia reduces respiratory capacity at 12 h and basal respiration after 48 h (42). Moreover, the metabolic profile is changed with alternative energy sources and an inhibition of the tricarboxylic acid cycle under hyperoxia (43). Finally, hyperoxia is associated with morphological damage and promotes mitochondrial fragmentation (44). In conclusion, there is strong evidence, that hyperoxia exerts direct effects on cell metabolism, energy generation, and structural integrity. However, we focused on microcirculatory changes in hemorrhagic shock states and could not show any beneficial or detrimental effect of local hyperoxia on microvascular parameters. Due to the minimal-invasive setup and the intent to avoid surgical trauma to preserve a mostly physiological microcirculation we were not able to measure local oxygen partial pressure for example by Clark like electrodes. While oxygen is able to cross epithelial borders, the gastric mucus layer may be an obstacle that could hamper gastric tissue protection by local oxygen application. In our experiments neither the gastric nor the oral oxygenation, where no mucus exists to impair transepithelial oxygen transport, improved after oxygen application. We conclude that local oxygen application fails to improve gastrointestinal oxygenation independently of any limitation by biological borders like gastric mucus.

In the second set of experiments, we investigated the impact of local CO_2_-application on gastrointestinal microcirculation and oxygenation. According to our previous results with systemic hypercapnia (11, 45) we could show that also locally applied CO_2_ significantly improves microvascular oxygenation at the oral and the gastric surface. Since microvascular oxygenation represents predominantly postcapillary oxygen saturation, an increase in microvascular oxygenation may be paralleled by a higher local oxygen reserve (23). Avoiding hypoxic states in the course of hemorrhage could preserve intestinal integrity and impede the development of multi organ failure and systemic inflammatory states. We were not able to show that an increase of microvascular oxygenation preserves intestinal barrier function assessed by plasmatic sucrose concentration after gastric application. During the experiments we could observe a gastroesophageal reflux that led to a wide range of inter-individual variability. Additionally, sucrose itself may contribute to an increase of intestinal permeability due to its high osmolarity (46). Therefore, results from our measurements of intestinal permeability have to be considered with care.

Higher values of postcapillary oxygen saturation can be a result of an increase in local oxygen supply due to precapillary vasodilatation, lower oxygen liberalization from hemoglobin molecules, an increase of oxygen diffusion distance as a result of microvascular shunting and finally differences of mitochondrial energy metabolism.

CO_2_ is reported to have vasodilatory properties through K^+^_*ATP*_-channels in coronary vessels (47), which might improve microvascular oxygen delivery through an increase in microvascular perfusion. However, Beck et al. could show that beneficial effects of acute hypercapnia on microcirculatory oxygenation are independent of K^+^_*ATP*_-channels in sepsis. Further, total microvascular blood flow did not change in our experiments when local hypercapnia was applied (10). The second crucial step of microvascular oxygen delivery is oxygen liberalization from hemoglobin molecules and the diffusion toward mitochondria. Hypercapnia and hypercapnic acidosis led to a right-shift of hemoglobin dissociation curve and a simplified oxygen liberalization. Therefore, oxygen liberalization is not expected to be hampered by local CO_2_-application. We did not observe a reduction of functional capillary density in hypercapnic animals as a sign of microvascular shunting and an increase in microvascular diffusion distance. Hampered oxygen liberalization as well as microvascular shunting led to an increased postcapillary oxygenation accompanied by cellular hypoxia and lactate generation. In the same manner, dysfunctional oxygen utilization in the mitochondria with an anaerobic energy metabolism is associated with a corresponding increase in lactate levels (48). Finally, there were no difference in lactate plasma levels between hypercapnic and normocapnic individuals undergoing hemorrhage. If cell metabolism is altered, especially a reduction of basal energy metabolism or an increase in mitochondrial effectivity without cellular hypoxia and mitochondrial failure would be compatible with our results. Direct effects of carbon dioxide on cell respiration and integrity were described in an experimental setting before (49, 50).

Like mentioned above, this study was not designed to describe mitochondrial function but functional and structural microcirculation. Microvascular oxygenation was paralleled by changes in microvascular flow characteristics at the oral surface. We could observe a more continuous microvascular blood flow under hypercapnia in hemorrhagic shock states represented by higher values of microvascular flow index (MFI). In a septic patient collective microcirculatory alterations were associated with high mortality (51–54), with the proportion of perfused vessel and microvascular flow index being the strongest predictors for a worse outcome (53, 54). Especially a compromised MFI was identified to be an independent predictor of mortality in a general ICU population (28, 29). Our results may implicate, that local hypercapnia is able to ameliorate microvascular oxygenation through a continuous blood flow within the microcirculation without any impact on total microvascular blood flow.

Hypercapnia is known to increase plasmatic vasopressin levels (55, 56). Further, hypercapnia as well as low-doses vasopressin are able to improve microvascular oxygenation under physiological conditions (45, 57). Those findings can be transferred into animals undergoing sepsis (58, 59). Whereas systemic hypercapnia is known to improve tissue oxygenation under adverse conditions like hemorrhage (11) and sepsis (9), the role of vasopressin in resuscitation bundles remain unclear. This study is pointing toward the participation of carbon dioxide in regional blood flow distribution. If this effect depends on local vasopressin release and signal transmission via vasopressin receptors has to be clarified. The V_1A_-receptor is reported to mediate a dose-dependent effect on gastric microcirculation under physiological conditions (57) and stabilizes intestinal oxygenation during hypercapnia in septic (60) and hemorrhagic (61) animals. If the protection of gastric and oral oxygenation by local hypercapnia is mediated by a V_1A_-receptor dependent increase in flow homogeneity and related to and improved mitochondrial respiration has to be evaluated in further studies.

Systemic side effects occur under systemic hypercapnia and could limit therapeutic approaches (62). For example, acute hemorrhage occurs in patients undergoing polytrauma, a collective that frequently suffers traumatic head injury (63). A hypercapnic precapillary vasodilation could increase intracerebral pressure (64) and proceed further neurological injury. Besides, low pH-values are leading to an insufficient hemostasis (65), which could be detrimental in the context of acute hemorrhage and major surgery with a high need for sufficient bleeding control. Beneficial effects of local hypercapnia were independent from systemic oxygen delivery, blood gases and pH-value in our experiments. Thus, local gas applications might provide safe options in the treatment of critically ill patients without any impact on systemic parameters.

One may assume, that local gas insufflation is an unsuitable therapeutic approach, due to the large intestinal surface and the corresponding gas volume needed to establish a gaseous atmosphere for all over intestinal protection. Therefore, the investigation of receptor related CO_2_-effects could be the next step and a cornerstone in the development of pharmacological tissue protection during acute hemorrhage. A direct interaction of carbon dioxide and vasopressin is described in different shock models and has to be further investigated (60, 61). Beyond the treatment of injured patients, small intestinal anastomosis dehiscence is a frequent complication after abdominal surgery (66) with the need for consecutive operations. Local CO_2_, when applicated endoscopically, could improve regional microcirculation, and oxygenation in intention to avoid anastomosis dehiscence. A beneficial effect of local carbon dioxide on wound healing is described in a wide context (67).

Microcirculatory alterations in critical illness are described to correlate within the vascular beds of the gastrointestinal tract when tonometry (68) or videomicroscopy (69) is used. However, our experiments showed a prolonged protective effect of local CO_2_-application on oral oxygenation compared to gastric measurements, which might be an effect of a variable density of receptors providing vasoconstriction. Schwartges et al. reported a concentration-dependent increase in gastric μHbO_2_ under systemic hypercapnia in normovolemic dogs (45). Despite, μHbO_2_ was not altered by local CO_2_-supply under physiological conditions, whereas the same amount CO_2_ was able to improve μHbO_2_ under hemorrhagic conditions in our experiments. Probably, the effect of local hypercapnia on mucosal microcirculation depends on the hemodynamic conditions.

## Conclusion

Hypercapnia is reported to exert beneficial effects on intestinal microcirculation. Since systemic side effects may limit therapeutic approaches in patients with multiple comorbidities, local therapeutic regimes could be used to enable the clinical transfer of therapeutic hypercapnia into a critically ill patient collective. We could demonstrate that local carbon dioxide application is able to improve gastric and oral postcapillary oxygen saturation during a mild hemorrhagic shock. This effect might be mediated by improved microvascular flow under local carbon dioxide application. This may implicate an increase of local oxygen reserve. The redistribution of available oxygen carriers leading to continuous blood flow profiles at the mucosal surfaces seems to be more important than total blood flow within the microcirculation. While local carbon dioxide application improved parameters of microcirculatory oxygen delivery without any effect on systemic hemodynamic variables and blood acidity, local oxygen supply did not influence postcapillary oxygen saturation, flow heterogeneity and systemic parameters. From these results we can conclude that local gas application is feasible without any systemic side effects in the chosen experimental model and might probably be applied safely, even when adverse conditions occur in critically ill patients.

## Data Availability Statement

The datasets presented in this study can be found in online repositories. The names of the repository/repositories and accession number(s) can be found in the article/Supplementary Material.

## Ethics Statement

The animal study was reviewed and approved by the local Animal Care and Use Committee (North Rhine-Westphalia State Agency for Nature, Environment and Consumer Protection, Recklinghausen, Germany).

## Author Contributions

SH: acquisition of data, analysis and interpretation of data, and drafting the article. RT: conception and design, acquisition of data, analysis and interpretation of data, and drafting the article. LW: acquisition of data, analysis and interpretation of data, and revising the article. AH and JS: analysis and interpretation of data, and revising the article. AW, IB, OP, and CV: conception and design, analysis and interpretation of data, and revising the article. EM: conception and design, acquisition of data, analysis and interpretation of data, and revising the article. All authors read and approved the final manuscript.

## Conflict of Interest

The authors declare that the research was conducted in the absence of any commercial or financial relationships that could be construed as a potential conflict of interest.

## Publisher’s Note

All claims expressed in this article are solely those of the authors and do not necessarily represent those of their affiliated organizations, or those of the publisher, the editors and the reviewers. Any product that may be evaluated in this article, or claim that may be made by its manufacturer, is not guaranteed or endorsed by the publisher.
