# Supplementary material for: Local Mucosal CO2 but Not O2 Insufflation Improves Gastric and Oral Microcirculatory Oxygenation in a Canine Model of Mild Hemorrhagic Shock
Source: Front Med (Lausanne). 2022 Apr 28;9:867298. doi: 10.3389/fmed.2022.867298 (PMC9096873; doi:10.3389/fmed.2022.867298)
Supplement: Supplementary file 1 [file Table_1.DOCX]

Suppl. Data Tab. 1: Microcirculatory variables in normo- (NO) and hyperoxic (HO) animals during physiological (-N) or hemorrhagic (-H) conditions. Microvascular oxygenation (µHbO_2_) in percentage [%] and microvascular flow (µflow) in arbitrary units [aU] were measured at the gastric and oral surface using reflectance spectrophotometry and laser Doppler flowmetry. Microvascular flow index (MFI), heterogeneity index (HGI), total vessel density (TVD) in [mm/mm^2^] and PVD in [mm/mm^2^] were evaluated by incident dark field imagine. One hour of acute hemorrhage is marked grey. Data are presented as mean ± SEM for n = 6 dogs. ^#^p<0.05 vs. baseline, ^*^p<0.05 vs. normoxic control group. 2-way ANOVA for repeated measurements followed by Bonferroni post hoc test.

| variables | group | 00:30 h | | |  | 01:00 h | | |  | 01.30 h | | |  | 02:00 h | | |  | 02:30 h | | |  | 03:00 h | | |  | 03:30 h | | |  |
| --- | --- | --- | --- | --- | --- | --- | --- | --- | --- | --- | --- | --- | --- | --- | --- | --- | --- | --- | --- | --- | --- | --- | --- | --- | --- | --- | --- | --- | --- |
| gastric µHbO_2_ [%] | NO-N | 78 | ± | 3 |  | 82 | ± | 2 |  | 81 | ± | 3 |  | 81 | ± | 3 |  | 78 | ± | 3 |  | 78 | ± | 2 |  | 76 | ± | 2 |  |
|  | HO-N | 77 | ± | 2 |  | 78 | ± | 2 |  | 77 | ± | 2 |  | 80 | ± | 1 |  | 79 | ± | 1 |  | 78 | ± | 1 |  | 79 | ± | 2 |  |
|  | NO-H | 77 | ± | 2 |  | 80 | ± | 2 |  | 82 | ± | 1 |  | 46 | ± | 8 | **#** | 48 | ± | 7 | **#** | 75 | ± | 2 |  | 79 | ± | 2 |  |
|  | HO-H | 78 | ± | 1 |  | 78 | ± | 2 |  | 77 | ± | 2 |  | 45 | ± | 6 | **#** | 48 | ± | 7 | **#** | 77 | ± | 4 |  | 79 | ± | 3 |  |
| gastric µflow [aU] | NO-N | 148 | ± | 15 |  | 176 | ± | 16 |  | 187 | ± | 17 |  | 178 | ± | 14 |  | 158 | ± | 17 |  | 156 | ± | 20 |  | 133 | ± | 16 |  |
|  | HO-N | 154 | ± | 15 |  | 194 | ± | 14 |  | 193 | ± | 19 |  | 190 | ± | 17 |  | 194 | ± | 27 |  | 167 | ± | 21 |  | 160 | ± | 27 |  |
|  | NO-H | 141 | ± | 16 |  | 153 | ± | 23 |  | 176 | ± | 14 |  | 149 | ± | 34 |  | 158 | ± | 24 |  | 170 | ± | 20 |  | 207 | ± | 29 |  |
|  | HO-H | 14 | ± | 11 |  | 203 | ± | 26 | **#** | 164 | ± | 16 |  | 151 | ± | 16 |  | 142 | ± | 17 |  | 196 | ± | 44 |  | 170 | ± | 29 |  |
| oral µHbO_2_ [%] | NO-N | 86 | ± | 2 |  | 81 | ± | 3 |  | 83 | ± | 2 |  | 82 | ± | 2 |  | 83 | ± | 3 |  | 80 | ± | 4 |  | 81 | ± | 3 |  |
|  | HO-N | 82 | ± | 1 |  | 81 | ± | 2 |  | 82 | ± | 2 |  | 83 | ± | 2 |  | 82 | ± | 1 |  | 82 | ± | 2 |  | 85 | ± | 2 |  |
|  | NO-H | 86 | ± | 1 |  | 82 | ± | 2 |  | 86 | ± | 1 |  | 46 | ± | 4 | **#** | 44 | ± | 6 | **#** | 85 | ± | 4 |  | 94 | ± | 2 | **#** |
|  | HO-H | 80 | ± | 1 |  | 79 | ± | 2 |  | 81 | ± | 3 |  | 43 | ± | 5 | **#** | 49 | ± | 4 | **#** | 86 | ± | 2 |  | 88 | ± | 2 |  |
| oral µfow [aU] | NO-N | 97 | ± | 15 |  | 96 | ± | 16 |  | 92 | ± | 9 |  | 97 | ± | 16 |  | 102 | ± | 18 |  | 131 | ± | 23 |  | 127 | ± | 11 |  |
|  | HO-N | 129 | ± | 27 |  | 98 | ± | 17 |  | 110 | ± | 20 |  | 115 | ± | 18 |  | 96 | ± | 16 |  | 103 | ± | 25 |  | 106 | ± | 8 |  |
|  | NO-H | 135 | ± | 24 |  | 147 | ± | 26 |  | 122 | ± | 17 |  | 54 | ± | 8 | **#** | 41 | ± | 9 | **#** | 149 | ± | 34 |  | 214 | ± | 28 | **#** |
|  | HO-H | 93 | ± | 13 |  | 114 | ± | 23 |  | 82 | ± | 11 |  | 23 | ± | 6 | **#** | 31 | ± | 8 | **#** | 91 | ± | 17 | ***** | 145 | ± | 15 | **#,*** |
| MFI | NO-N | 2.9 | ± | 1.1 |  | 2.7 | ± | 1.2 |  | 2.7 | ± | 1.1 |  | 2.7 | ± | 1.1 |  | 2.6 | ± | 1.2 |  | 2.8 | ± | 1.1 |  | 2.5 | ± | 1.2 |  |
|  | HO-N | 2.7 | ± | 1.1 |  | 2.8 | ± | 1.1 |  | 2.5 | ± | 1.1 |  | 2.8 | ± | 1.1 |  | 2.7 | ± | 1.1 |  | 2.6 | ± | 1.2 |  | 2.6 | ± | 1.1 |  |
|  | NO-H | 2.5 | ± | 1 |  | 2.6 | ± | 1.1 |  | 2.7 | ± | 1.1 |  | 1.4 | ± | 0.7 | **#** | 1.6 | ± | 1 | **#** | 2.8 | ± | 1.1 |  | 2.9 | ± | 1.2 |  |
|  | HO-H | 2.8 | ± | 1.1 |  | 2.6 | ± | 1.2 |  | 2.5 | ± | 1.1 |  | 1.5 | ± | 0.8 | **#** | 1.4 | ± | 0.8 | **#** | 2.7 | ± | 1.2 |  | 2.7 | ± | 1.1 |  |
| HGI | NO-N | 0.4 | ± | 0.2 |  | 0.4 | ± | 0.1 |  | 0.4 | ± | 0.2 |  | 0.4 | ± | 0.2 |  | 0.4 | ± | 0.1 |  | 0.4 | ± | 0.2 |  | 0.4 | ± | 0.1 |  |
|  | HO-N | 0.4 | ± | 0.2 |  | 0.4 | ± | 0.2 |  | 0.8 | ± | 0.1 |  | 0.4 | ± | 0.2 |  | 0.4 | ± | 0.3 |  | 0.4 | ± | 0.1 |  | 0.4 | ± | 0.2 |  |
|  | NO-H | 0.4 | ± | 0.2 |  | 0.4 | ± | 0.2 |  | 0.4 | ± | 0.2 |  | 1.4 | ± | 0.5 | **#** | 1.3 | ± | 0.3 | **#** | 0.4 | ± | 0.2 |  | 0.4 | ± | 0.1 |  |
|  | HO-H | 0.4 | ± | 0.2 |  | 0.4 | ± | 0.1 |  | 0.4 | ± | 0.2 |  | 0.7 | ± | 0.4 | **#** | 1.5 | ± | 0 | **#** | 0.4 | ± | 0.1 |  | 0.4 | ± | 0.2 |  |
| TVD [mm/mm^2^] | NO-N | 17 | ± | 1 |  | 17 | ± | 1 |  | 17 | ± | 1 |  | 17 | ± | 1 |  | 16 | ± | 1 |  | 17 | ± | 1 |  | 17 | ± | 1 |  |
|  | HO-N | 18 | ± | 1 |  | 18 | ± | 1 |  | 18 | ± | 1 |  | 18 | ± | 1 |  | 18 | ± | 1 |  | 19 | ± | 1 |  | 18 | ± | 1 |  |
|  | NO-H | 16 | ± | 1 |  | 16 | ± | 1 |  | 16 | ± | 1 |  | 13 | ± | 1 | **#** | 13 | ± | 1 | **#** | 18 | ± | 1 |  | 18 | ± | 1 |  |
|  | HO-H | 18 | ± | 1 |  | 17 | ± | 1 |  | 17 | ± | 1 |  | 15 | ± | 1 | **#,*** | 14 | ± | 1 | **#** | 17 | ± | 1 |  | 18 | ± | 1 |  |
| PVD [mm/mm^2^] | NO-N | 6 | ± | 1 |  | 6 | ± | 1 |  | 5 | ± | 1 |  | 4 | ± | 1 |  | 5 | ± | 1 |  | 5 | ± | 1 |  | 6 | ± | 1 |  |
|  | HO-N | 6 | ± | 1 |  | 6 | ± | 1 |  | 6 | ± | 1 |  | 6 | ± | 1 |  | 5 | ± | 1 | **#** | 6 | ± | 2 |  | 7 | ± | 2 |  |
|  | NO-H | 4 | ± | 1 |  | 4 | ± | 1 |  | 5 | ± | 1 |  | 2 | ± | 1 |  | 2 | ± | 1 |  | 9 | ± | 1 | **#,*** | 8 | ± | 1 | **#** |
|  | HO-H | 6 | ± | 1 |  | 5 | ± | 1 |  | 5 | ± | 1 |  | 3 | ± | 1 |  | 3 | ± | 1 |  | 6 | ± | 1 |  | 7 | ± | 1 |  |
