# Supplementary material for: Local Mucosal CO2 but Not O2 Insufflation Improves Gastric and Oral Microcirculatory Oxygenation in a Canine Model of Mild Hemorrhagic Shock
Source: Front Med (Lausanne). 2022 Apr 28;9:867298. doi: 10.3389/fmed.2022.867298 (PMC9096873; doi:10.3389/fmed.2022.867298)
Supplement: Supplementary file 2 [file Table_2.DOCX]

Suppl. Data Tab. 2. Macrocirculatory variables and blood gas analysis in normo- (NO) and hyperoxic (HO) animals during physiological (-N) or hemorrhagic (-H) conditions. Systemic oxygen delivery (DO_2_) in [ml/kg/min], cardiac output (CO) in [ml/kg/min], mean arterial pressure (MAP) in [mmHg], pH-values, arterial carbon dioxide pressure (p_a_CO_2_) in [mmHg], base excess (BE) in [mmol/l], lactate concentration in [mmol/l] and arterial oxygen pressure (p_a_O_2_) in [mmHg]. One hour of acute hemorrhage is marked grey. Data are presented as mean ± SEM for n = 6 dogs. ^#^p<0.05 vs. baseline, ^*^p<0.05 vs. normoxic control group. 2-way ANOVA for repeated measurements followed by Bonferroni post hoc test.

| variables | group | 00:30 h | | |  | 01:00 h | | |  | 01.30 h | | |  | 02:00 h | | |  | 02:30 h | | |  | 03:00 h | | |  | 03:30 h | | |  |
| --- | --- | --- | --- | --- | --- | --- | --- | --- | --- | --- | --- | --- | --- | --- | --- | --- | --- | --- | --- | --- | --- | --- | --- | --- | --- | --- | --- | --- | --- |
| DO_2_ [ml/kg/min] | NO-N | 13 | ± | 2 |  | 13 | ± | 2 |  | 13 | ± | 2 |  | 13 | ± | 2 |  | 13 | ± | 1 |  | 12 | ± | 1 |  | 13 | ± | 1 |  |
|  | HO-N | 13 | ± | 1 |  | 13 | ± | 1 |  | 13 | ± | 1 |  | 13 | ± | 1 |  | 13 | ± | 1 |  | 13 | ± | 1 |  | 13 | ± | 1 |  |
|  | NO-H | 13 | ± | 1 |  | 13 | ± | 1 |  | 13 | ± | 1 |  | 7 | ± | 1 | **#** | 8 | ± | 0 | **#** | 12 | ± | 1 |  | 13 | ± | 1 |  |
|  | HO-H | 13 | ± | 1 |  | 13 | ± | 1 |  | 13 | ± | 1 |  | 8 | ± | 1 | **#** | 8 | ± | 0 | **#** | 14 | ± | 1 |  | 14 | ± | 1 | **#** |
| CO [ml/kg/min] | NO-N | 83 | ± | 7 |  | 81 | ± | 7 |  | 82 | ± | 7 |  | 78 | ± | 7 |  | 79 | ± | 6 |  | 77 | ± | 7 |  | 79 | ± | 6 |  |
|  | HO-N | 82 | ± | 6 |  | 79 | ± | 5 |  | 79 | ± | 6 |  | 78 | ± | 6 |  | 78 | ± | 6 |  | 79 | ± | 6 |  | 78 | ± | 6 |  |
|  | NO-H | 82 | ± | 5 |  | 79 | ± | 4 |  | 78 | ± | 3 |  | 43 | ± | 3 | **#** | 51 | ± | 2 | **#** | 78 | ± | 3 |  | 84 | ± | 4 |  |
|  | HO-H | 83 | ± | 5 |  | 83 | ± | 6 |  | 83 | ± | 5 |  | 48 | ± | 3 | **#** | 53 | ± | 2 | **#** | 87 | ± | 4 | ***** | 91 | ± | 5 | **#,*** |
| MAP [mmHg] | NO-N | 61 | ± | 2 |  | 64 | ± | 2 |  | 65 | ± | 2 |  | 65 | ± | 3 |  | 67 | ± | 2 | **#** | 68 | ± | 3 | **#** | 69 | ± | 3 | **#** |
|  | HO-N | 61 | ± | 1 |  | 64 | ± | 1 |  | 66 | ± | 2 | **#** | 66 | ± | 2 | **#** | 66 | ± | 2 | **#** | 67 | ± | 2 | **#** | 68 | ± | 2 | **#** |
|  | NO-H | 61 | ± | 1 |  | 64 | ± | 1 |  | 65 | ± | 2 | **#** | 50 | ± | 2 | **#** | 59 | ± | 1 |  | 77 | ± | 1 | **#** | 69 | ± | 1 | **#** |
|  | HO-H | 65 | ± | 3 |  | 66 | ± | 2 |  | 71 | ± | 3 | **#,*** | 50 | ± | 2 | **#** | 58 | ± | 2 | **#** | 80 | ± | 2 | **#** | 72 | ± | 2 | **#** |
| pH | NO-N | 7.40 | ± | 0.02 |  | 7.39 | ± | 0.02 |  | 7.39 | ± | 0.01 |  | 7.38 | ± | 0.01 | **#** | 7.37 | ± | 0.02 | **#** | 7.36 | ± | 0.02 | **#** | 7.36 | ± | 0.02 | **#** |
|  | HO-N | 7.39 | ± | 0.01 |  | 7.39 | ± | 0.01 |  | 7.39 | ± | 0.01 |  | 7.38 | ± | 0.01 | **#** | 7.38 | ± | 0.01 | **#** | 7.37 | ± | 0.02 | **#** | 7.37 | ± | 0.02 | **#** |
|  | NO-H | 7.39 | ± | 0.01 |  | 7.38 | ± | 0.01 |  | 7.38 | ± | 0.01 |  | 7.30 | ± | 0.02 | **#** | 7.31 | ± | 0.02 | **#** | 7.35 | ± | 0.02 | **#** | 7.35 | ± | 0.01 | **#** |
|  | HO-H | 7.39 | ± | 0.01 |  | 7.39 | ± | 0.01 |  | 7.38 | ± | 0.01 |  | 7.31 | ± | 0.02 | **#** | 7.32 | ± | 0.01 | **#,*** | 7.37 | ± | 0.01 | **#,*** | 7.38 | ± | 0.01 | **#,*** |
| p_a_CO_2_ [mmHg] | NO-N | 36 | ± | 1 |  | 37 | ± | 1 |  | 36 | ± | 0 |  | 36 | ± | 1 |  | 37 | ± | 1 |  | 37 | ± | 1 |  | 38 | ± | 1 | **#** |
|  | HO-N | 36 | ± | 1 |  | 37 | ± | 1 |  | 37 | ± | 1 |  | 37 | ± | 1 |  | 37 | ± | 1 |  | 37 | ± | 1 |  | 37 | ± | 1 |  |
|  | NO-H | 37 | ± | 1 |  | 37 | ± | 1 |  | 37 | ± | 1 |  | 42 | ± | 1 | **#** | 41 | ± | 1 | **#** | 37 | ± | 1 |  | 38 | ± | 1 |  |
|  | HO-H | 36 | ± | 1 |  | 36 | ± | 1 |  | 37 | ± | 1 |  | 41 | ± | 1 | **#** | 39 | ± | 1 | **#,*** | 36 | ± | 1 |  | 37 | ± | 1 |  |
| BE [mmol/l] | NO-N | -2.2 | ± | 0.6 |  | -2.5 | ± | 0.5 |  | -3.1 | ± | 0.7 | **#** | -3.2 | ± | 0.5 | **#** | -3.6 | ± | 0.6 | **#** | -3.7 | ± | 0.8 | **#** | -3.8 | ± | 0.8 | **#** |
|  | HO-N | -2.5 | ± | 0.2 |  | -2.6 | ± | 0.2 |  | -2.9 | ± | 0.2 |  | -3.2 | ± | 0.4 |  | -3.3 | ± | 0.4 | **#** | -3.4 | ± | 0.5 | **#** | -3.4 | ± | 0.6 | **#** |
|  | NO-H | -2.5 | ± | 0.3 |  | -3.0 | ± | 0.4 |  | -3.3 | ± | 0.5 | **#** | -5.3 | ± | 0.7 | **#** | -5.3 | ± | 0.7 | **#** | -4.5 | ± | 0.5 | **#** | -4.1 | ± | 0.6 | **#** |
|  | HO-H | -2.5 | ± | 0.4 |  | -2.7 | ± | 0.3 |  | -3.2 | ± | 0.3 |  | -5.0 | ± | 0.5 | **#** | -5.2 | ± | 0.8 | **#** | -3.9 | ± | 0.3 | **#** | -3.4 | ± | 0.3 | **#** |
| lactate [mmol/l] | NO-N | 1.0 | ± | 0.2 |  | 1.2 | ± | 0.2 |  | 1.5 | ± | 0.1 |  | 1.9 | ± | 0.2 | **#** | 2.1 | ± | 0.3 | **#** | 2.2 | ± | 0.4 | **#** | 2.3 | ± | 0.4 | **#** |
|  | HO-N | 1.0 | ± | 0.2 |  | 1.1 | ± | 0.2 |  | 1.4 | ± | 0.2 |  | 1.6 | ± | 0.2 | **#** | 1.7 | ± | 0.2 | **#** | 1.8 | ± | 0.3 | **#** | 1.9 | ± | 0.2 | **#** |
|  | NO-H | 0.8 | ± | 0.1 |  | 1.2 | ± | 0.2 |  | 1.4 | ± | 0.2 | **#** | 2.0 | ± | 0.3 | **#** | 2.0 | ± | 0.3 | **#** | 1.8 | ± | 0.3 | **#** | 1.8 | ± | 0.3 | **#** |
|  | HO-H | 1.1 | ± | 0.1 |  | 1.3 | ± | 0.1 |  | 1.6 | ± | 0.2 | **#** | 1.9 | ± | 0.2 | **#** | 1.8 | ± | 0.2 | **#** | 1.6 | ± | 0.2 | **#** | 1.6 | ± | 0.2 |  |
| p_a_O_2_ [mmHg] | NO-N | 147 | ± | 4 |  | 151 | ± | 3 |  | 154 | ± | 5 |  | 153 | ± | 3 |  | 151 | ± | 5 |  | 155 | ± | 4 | **#** | 157 | ± | 4 | **#** |
|  | HO-N | 148 | ± | 4 |  | 148 | ± | 3 |  | 152 | ± | 3 |  | 154 | ± | 4 |  | 156 | ± | 4 | **#** | 154 | ± | 4 |  | 155 | ± | 3 | **#** |
|  | NO-H | 146 | ± | 4 |  | 149 | ± | 4 |  | 152 | ± | 4 |  | 137 | ± | 3 | **#** | 143 | ± | 3 |  | 157 | ± | 2 | **#** | 157 | ± | 3 | **#** |
|  | HO-H | 143 | ± | 4 |  | 145 | ± | 3 |  | 147 | ± | 3 |  | 133 | ± | 2 | **#** | 139 | ± | 3 |  | 152 | ± | 2 | **#** | 154 | ± | 3 | **#** |
