# Supplementary material for: Local Mucosal CO2 but Not O2 Insufflation Improves Gastric and Oral Microcirculatory Oxygenation in a Canine Model of Mild Hemorrhagic Shock
Source: Front Med (Lausanne). 2022 Apr 28;9:867298. doi: 10.3389/fmed.2022.867298 (PMC9096873; doi:10.3389/fmed.2022.867298)
Supplement: Supplementary file 3 [file Table_3.DOCX]

Suppl. Data Tab. 3: Plasmatic sucrose concentration [pmol/µl] in normo- (NC) and hypercapnic (HC) and normo- (NO) and hyperoxic (HO) animals during physiological (-N) or hemorrhagic (-H) conditions. One hour of acute hemorrhage is marked grey. Data are presented as mean ± SEM for n = 6 dogs. ^#^p<0.05 vs. baseline. 2-way ANOVA for repeated measurements followed by Bonferroni post hoc test.

| variables | group | 00:30 h | | |  | 01:00 h | | |  | 01.30 h | | |  | 02:00 h | | |  | 02:30 h | | |  |
| --- | --- | --- | --- | --- | --- | --- | --- | --- | --- | --- | --- | --- | --- | --- | --- | --- | --- | --- | --- | --- | --- |
| sucrose [pmol/µl] | NC-N | 1.7 | ± | 0.63 |  | 1.7 | ± | 0.63 |  | 1.7 | ± | 0.63 |  | 0.2 | ± | 0.11 |  | 2.3 | ± | 1.47 |  |
|  | HC-N | 1.2 | ± | 0.4 |  | 1.2 | ± | 0.4 |  | 1.2 | ± | 0.4 |  | 0.1 | ± | 0.09 |  | 1 | ± | 0.6 |  |
|  | NC-H | 1 | ± | 0.36 |  | 1 | ± | 0.36 |  | 1 | ± | 0.36 |  | 1.8 | ± | 1.3 |  | 1.3 | ± | 0.59 |  |
|  | HC-H | 0.2 | ± | 0.11 |  | 0.2 | ± | 0.11 |  | 0.2 | ± | 0.11 |  | 1.8 | ± | 1.1 |  | 1 | ± | 0.62 |  |
|  |  |  |  |  |  |  |  |  |  |  |  |  |  |  |  |  |  |  |  |  |  |
|  | NO-N | 1.7 | ± | 0.63 |  | 1.7 | ± | 0.63 |  | 1.7 | ± | 0.63 |  | 0.2 | ± | 0.11 |  | 2.3 | ± | 1.47 | # |
|  | HO-N | 1 | ± | 0.36 |  | 1 | ± | 0.36 |  | 1 | ± | 0.36 |  | 1.8 | ± | 1.3 |  | 1.3 | ± | 0.59 |  |
|  | NO-H | 1.2 | ± | 0.4 |  | 1.2 | ± | 0.4 |  | 1.2 | ± | 0.4 |  | 0.1 | ± | 0.09 |  | 1 | ± | 0.6 | # |
|  | HO-H | 0.2 | ± | 0.11 |  | 0.2 | ± | 0.11 |  | 0.2 | ± | 0.11 |  | 1.8 | ± | 1.1 |  | 1 | ± | 0.62 |  |
