# Supplementary figures and images for: Local Mucosal CO2 but Not O2 Insufflation Improves Gastric and Oral Microcirculatory Oxygenation in a Canine Model of Mild Hemorrhagic Shock
Source: Front Med (Lausanne). 2022 Apr 28;9:867298. doi: 10.3389/fmed.2022.867298 (PMC9096873; doi:10.3389/fmed.2022.867298)

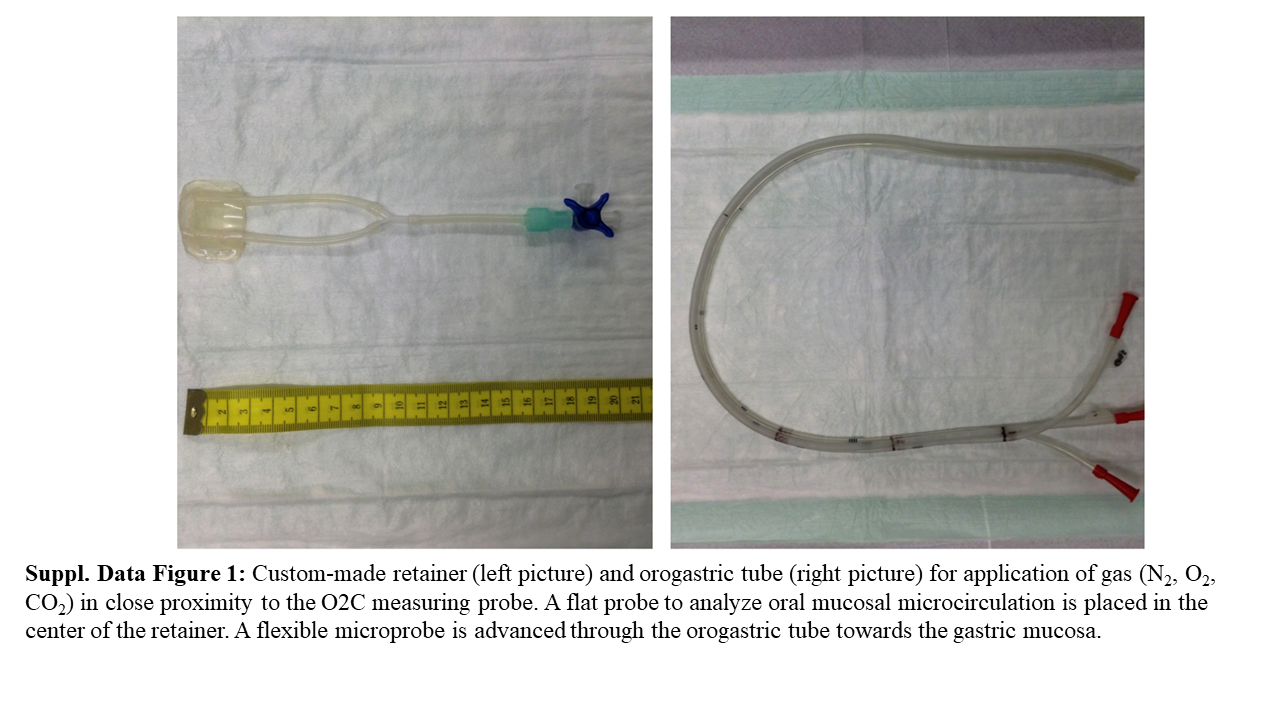

Supplement: Supplementary file 4 [file Image_1.tif]
